# Interference of Overlapping Insect Vibratory Communication Signals: An *Eushistus heros* Model

**DOI:** 10.1371/journal.pone.0130775

**Published:** 2015-06-22

**Authors:** Andrej Čokl, Raul Alberto Laumann, Alenka Žunič Kosi, Maria Carolina Blassioli-Moraes, Meta Virant-Doberlet, Miguel Borges

**Affiliations:** 1 Department of Entomology, National Institute of Biology, Ljubljana, Slovenia; 2 Semiochemical Laboratory, EMBRAPA Genetic Resources and Biotechnology, Brasilia, Brazil; University of Tours, FRANCE

## Abstract

Plants limit the range of insect substrate-borne vibratory communication by their architecture and mechanical properties that change transmitted signal time, amplitude and frequency characteristics. Stinkbugs gain higher signal-to-noise ratio and increase communication distance by emitting narrowband low frequency vibratory signals that are tuned with transmission properties of plants. The objective of the present study was to investigate hitherto overlooked consequences of duetting with mutually overlapped narrowband vibratory signals. The overlapped vibrations of the model stinkbug species *Eushistus heros*, produced naturally or induced artificially on different plants, have been analysed. They represent female and male strategies to preserve information within a complex masked signal. The brown stinkbugs *E*. *heros* communicate with species and gender specific vibratory signals that constitute characteristic duets in the calling, courtship and rivalry phases of mating behaviour. The calling female pulse overlaps the male vibratory response when the latency of the latter is shorter than the duration of the female triggering signal or when the male response does not inhibit the following female pulse. Overlapping of signals induces interference that changes their amplitude pattern to a sequence of regularly repeated pulses in which their duration and the difference between frequencies of overlapped vibrations are related inversely. Interference does not occur in overlapped narrow band female calling pulses and broadband male courtship pulse trains. In a duet with overlapped signals females and males change time parameters and increase the frequency difference between signals by changing the frequency level and frequency modulation pattern of their calls.

## Introduction

Exchange of information by vibratory signals transmitted through plants is an important element of multimodal communication during mating of stinkbugs [1, 2] and most other insect groups [3]. On plants, insects use low frequency, corrected bending waves [4] characterized, among other factors, by frequency-dependent propagation velocity, low attenuation and non-linear decrease of amplitude with distance [5]. Stinkbugs of the subfamily Pentatominae tune the group-characteristic frequency of the vibratory signal emission and sensory systems [2]to the low-pass filtering properties of their host plants [6, 7, 8]. The dominant frequency of communication signals produced by abdomen tremulation ranges between 60 and 190 Hz [9] and their leg vibratory receptors [10] are tuned to frequencies below 300 Hz [11, 12, 13]. Communication through a narrow frequency window optimizes the discrimination of signals from environmental noise [14] on the one hand but, on the other, limits the potential range of species and gender specificity to the time and amplitude pattern parameters of vibratory signals [15]. Additionally, overlapping of signals decreases the effectiveness of mate recognition and its location on a plant [14]. Duetting mates generally emit spectrally similar signals in intervals between the consecutive partner’s calls by adapting their duration and repetition time. When different ranges of variation of signal time parameters prevent their adaptation, signals overlap and duetting mates are faced with the problem of contrasting and preserving their own information within complex vibration. This phenomenon has been under-investigated in Pentatomidae and other insect groups. The song repertoire of *Eushistus heros* (F.) (Heteroptera: Pentatomidae) was first recorded from couples mating on a loudspeaker membrane [16]. The authors described two female (FS-1, FS-2) and four different male (MS-1, MS-2, MS-3, MRS) songs. The FS-1 song was emitted as the first song in a calling duet with a male and the FS-2 song was recorded in the courtship phase of mating behaviour. MS-1 was triggered by female calling (FS-1) and MS-2 appeared in connection with FS-2 or as precursors to FS-1/MS-1 duets. The other two male songs were emitted during male rivalry (MRS) or prior to copulation attempt (MS-3). We have selected *Euschistus heros* (F.) as the model test species because the study conducted on the loudspeaker [16] revealed irregularly repeated vibratory signals of complex amplitude and frequency modulated pattern, indicating their possible origin in masked female and male emissions. Information on insect strategies to minimize the negative effect of signal overlapping is lacking and the role of frequency has not been investigated in this context. Two studies on Pentatomidae species support the hypothesis that modification of frequency characteristics of vibratory signals is involved as the reaction to masked emissions. *Nezara viridula* (L.) females change the dominant frequency of their calling song signals when they are overlapped by pure tone vibration of frequency differences less than 10 Hz [17] while *Piezodorus lituratus* (Fabricius) males respond by different songs to playback stimulation with the conspecific male rival song of modified frequency characteristics [18]. In the present study we have reinvestigated the vibratory songs emitted by *E*. *heros* on a plant, with special attention to overlapped female and male vibratory emissions. We analyzed the time and frequency characteristics of overlapped signals in natural conditions and by playback experiments with pre-recorded vibratory emissions and pure tones. We proposed the hypothesis that overlapping of narrow-band low frequency vibrations induces interference that consequently changes the amplitude modulation pattern of involved signals. We used pre-recorded signals and pure tones to exclude the active role of singing insects in this process. Finally we focused our attention to female and male reactions to avoid or minimize the effect of overlapping by modulation of signal time and frequency parameters. The objective of the present study is to confirm or reject the hypotheses that vibratory signals of *E*. *heros* often overlap, that overlapping induces interference and that in such a situation females and males react by changing time and frequency characteristics of masked (= overlapped) emissions.

## Materials and Methods

### Plants and animals

All experiments were conducted on adult colony reared *E*. *heros*. It is not an endangered or protected species so that no specific permits are required for their collection. The colony started from nymphs and adults collected on soybean fields near Brasília (15°47’37”S–47°52’57”W) that are not privately owned or environmentally protected. Virgin male and female adults were separated two days after the final moult and reared in an environmental room (26 ± 1°C, 60 ± 10% RH, 14:10 h LD photoperiod under fluorescent lights of 40W) in plastic cages of 26 cm height and 22 cm diameter. They were fed on raw peanut seeds (*Arachis hypogaea* L.), green beans (*Phaseolus vulgaris* L.) and sunflower seeds (*Helianthus annuus* L.). To ensure sexual maturity, experiments were conducted with 15–25 day old adults [19]. Each male or female was tested only once. Soybean *Glycine max* (L.*)* (N = 7), pigeonpea *Cayanus cayan* (L.) (N = 3) and common bean *Phaseolus vulgaris* L. (N = 1) were used as test plants. The plants were cultivated at EMBRAPA fields in Brasilia and potted in soil in plastic pots of 15–20 cm diameter. The length of seven test soybean plants’ stems ranged from 16 to 27 cm. Each of the two lower soybean leaves (4x3 cm) was inserted to the stem 12–18 cm above the soil by a 2–3 cm stalk. At the top of the stem two side (3–6 cm) and a middle (1 cm) stalk carried 3 leaves each with dimensions of 6x4 cm for the side leaves and 2x1 cm for the middle one. The length of three pigeonpea test plants’ stems ranged from 33 to 41 cm. Eight to eleven stalks of 4–6 cm length each branched from the stem with the lowest one inserted to the stem 17–19 cm above the soil. The distance between stem-stalk crossings ranged between 2 and 4 cm. Each stalk carried three leaves (6–8 x 2–3 cm). The stem of the test common bean plant was 8 cm long and two 5 cm stalks branched from it 6 cm above the soil; each stalk carried one (8x7 cm) leaf at its end. At the top, two side (7 and 10 cm) and a middle (1 cm) stalk with 3 leaves each branched from the stem. Their dimensions ranged from 8 to 11 x 5 to 6 cm for leaves of the side and 1 x 1 cm for those of the middle stalk.

### Recording and playback

Vibrations of the plant were recorded by a laser vibrometer (PDV-100, Polytec, Waldbronn, Germany) with the beam oriented perpendicular to the stem surface and focused 4 to 10.5 cm above the soil. To obtain stronger reflection, a small piece of reflecting tape (> 1 mm^2^ surface area) was glued to the recording point. Signals were digitized and stored via a sound card (24-bit, 96-kHz, 100-dB signal-to-noise ratio, Sound Blaster Extigy, Creative Laboratories Inc., Milpitas, CA, U.S.A.) using Cool Edit Pro 2.0 software (Adobe Systems Inc., San Jose, CA, U.S.A.). Values of frequency, time and amplitude of the recorded and digitized signals were measured using Sound Forge 6.0 software (Sonic Foundry Inc., Madison, WI, U.S.A.). Plants were vibrated by the cone screwed firmly to the top of the electrodynamic mini-shaker (4810, Bruel & Kjaer, Bruel & Kjaer, Naerum, Denmark) and attached by its tip to the stem 4–12 cm above the soil. To analyze the effect of pure tones on artificially induced signals the plant was vibrated by the second mini-shaker attached on the stem by the tip of the cone 5 cm above the point of vibration by the first exciter. Play-back programs were synthesized by the Cool Edit Pro 2.0 (see above) computer program. Pure tones of 75, 100, 125, 150 and 200 Hz were applied, either as continuous vibrations or as a sequence of fused, one second long pulses that frequency increased from 75 Hz of the first to 200 Hz of the last one. Pure tones were also used to get numerical data on relation between frequency difference of overlapped vibrations and duration of pulses induced by interference. The stimulus sequence of conspecific male first song (MS-1) pulses consisted of 5 signals that were recorded in the present study by a laser vibrometer from the bean. Stimulus MS-1 (N = 5), dominant frequency (115.8 ± 2.1 Hz), duration (6092.6 ± 775.3 ms) and repetition time (11317.0 ± 1045.3 ms) values corresponded to the range of those shown in Table 1. MS-1 playback signals were reproduced at mean velocities between 0.65 ± 0.09 and 14.05 ± 0.56 mm/s and continuous pure tones between 0.12 and 2.41 mm/s. Velocities of playback stimuli and continuous pure tones corresponded to values of naturally emitted vibratory signals recorded on the plant. According to the distance between the emitter and recording point the velocity of naturally emitted signals ranged between 0.5 and 15 mm/s.

**Table 1:** Temporal and frequency properties of *Eushistus heros* FS-1, MS-1, MS-2 and MRS signals.

Song type N/n	FS-1a 20/5	FS-1b 20/5	MS-1 20/8	MS-2 50/6	MRS 20/6
**Duration (ms)**	817.4±67.8 1381.5±150.3	924.5±186.0 1605±295.0	6839.2±1872.6 (n = 153, N = 8)	57.9±9.1 130.5±19.3	735.2±225.1 1403.8±382.4
**Repetition time (ms)**	2748.0±718.4 5014.0±1607–3	2475.4±328.7 3097.6±534.5	16104.5±9103 34069.5±8859	140.7±39.5 288.8±50.7	998.0±185.0 1853.1±492.8
**Dominant freq. (Hz)**	111.2±1.7 129.7±3.9	107.4±4.1 134.4±1.7	106.9±3.6 158.7±3.7	105.0±3.3 153.3±10.2	108.3±3.0 136.4±7.1
**Freq. FM-start (Hz)**	102.3±3.3 119.7±8.7	105.4±2.3 133.4±3.2	118.8±3.6 190.5±4.9	-	143.7±7.2 167.2±8.7
**Freq. FM-middle (Hz)**	-	-	-	-	107.8±2.4 126.6±4.0
**Freq. FM-end (Hz)**	127.1±2.5 152.0±5.1	119.2±6.0 144.7±4.5	101.5±2.9 153.5±4.8	-	86.1±4.3 101.0±6.1
**FM start-end (Hz)**	19.8±8.0 35.5±5.3	8.8±5.5 14.0±5.1	15.4±5.5 44.9±6.6	-	58.0±8.8 71.7±9.6
**FM/1000 ms Hz/1000 ms**	8.62±5.82 31.57±9.24	6.13±4.41 15.49±6.63	2.4±0.8 6.7±2.0	-	47.1±9.0 87.7±28.7

Minimal and maximal means with standard deviations are shown when differences among individuals were significant: FS-1a (One-way ANOVA, F>11.27, df = 4, P<0.0001), FS-1b (One-way ANOVA, F>3.389, df = 4, P<0.05), MS-1 duration (One-way ANOVA, F = 2.055, df = 7, P = 0.0521), MS-1 others (One-way ANOVA, F>8.59, df = 7, P<0,0001), MS-2 (One-way ANOVA, F>8.598, df = 3, P<0.0001), MRS (One-way ANOVA, F>6.71, df = 4, P<0.0001). N, number of signals analyzed for each individual; n, number of animals; FS-1a = female first song in duet with MS-1; FS-1b = female first song in duet with MS-2; MS-1 = first male song; MS-2 = second male song; MRS = male rival song; Freq = frequency, FM = frequency modulation.

### Experimental set-up and protocol

All experiments were conducted in a sound and vibration insulated room at the EMBRAPA Genetic Resources and Biotechnology, Brasilia-DF at room temperature (22–24°C), 45–60% relative humidity and room light conditions. Experiments were started by placing a pair of *E*. *heros* adults on different leaves of a selected plant. To induce rival singing the second male was added on the leaf opposite to the one on which a male and a female were alternating. Behaviour and emission of vibratory signals were monitored and recorded for 15 minutes for 35 pairs (28 couples copulated) and for 17 pairs in the presence of an added male (15 couples copulated). The same protocol was used when the effect of a pure tone plant vibration on communication of a couple was tested; for each frequency we tested 4 pairs.

### Terminology and data analysis

According to the order of song appearance in a duetting couple we classified them as the female or male first song (FS-1 and MS-1), the male second song (MS-2) and the male rival song (MRS). Vibratory signals that constitute the songs are described by their time (duration and repetition time of pulses and/or pulse trains) and frequency (dominant frequency and frequency modulation quantified by the frequency difference per time) characteristics. Pulses are defined as unitary homogenous parcels of vibrations of finite duration [20] and pulse trains as pulses arranged in groups with distinct start and end. Pulse duration was measured between signal onset and offset determined as the point where the amplitude increased above and decreased below the noise level. The repetition time was measured between onsets of two sequential signals. Frequency spectra and sonograms were obtained by the use of Sound Forge 6.0 (32768 FTE, 99% FFT overlap, Blackman-Harris smoothing window, slices displayed 1 and 9000 sonogram resolution) software. Frequency modulation (FM) of a signal was expressed as the dominant frequency difference (determined at signal 500 ms long start and end) divided by its duration. In MRS we described the FM pattern also by the dominant frequency of the middle 500 ms long part between values determined at 500 ms signal’s start and end. Velocity (vector quantity that specifies time rate of displacement) was measured at the pulse peak amplitude by the laser vibrometer set at maximum sensitivity. Interference is defined as the phenomenon that occurs when two or more wave trains of the same or similar frequency overlap, creating an interference pattern that looks different from either of the original wave patterns. Interference changes the continuous wave pattern into a sequence of fused pulses. The relation between the duration of pulses produced by interference and the frequency difference was expressed by the difference of dominant frequencies and mean pulse duration within a masked region. Data are represented as means (± SD) together with the number of pulses analyzed (N) and the number of animals (n) from which the pulses were obtained. When statistically significant differences between groups (individuals) of values existed, data are represented as minimum and maximum mean values. Two-tailed Student’s *t*-test and analysis of variance (One-way ANOVA) were used for statistical data processing.

## Results

### Calling, courtship and rivalry duets on a plant

**Fig 1:**
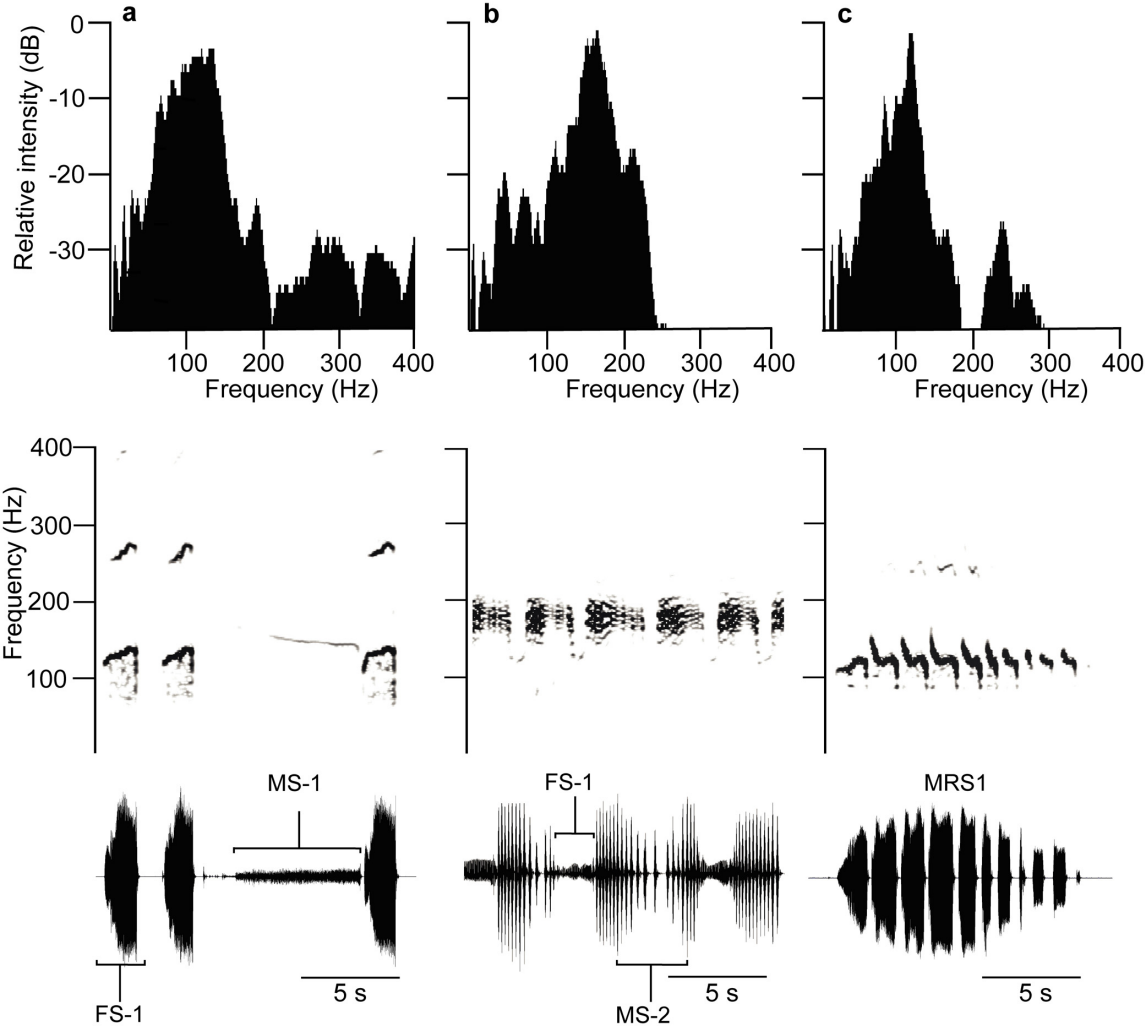
Spectra (upper), sonograms (middle) and oscillograms (lower) of *Eushistus heros* vibratory communication signals. a: FS-1 and MS-1 pulses in a calling duet, b: FS-1 pulses and MS-2 pulse trains in a courtship duet, c: MRS pulse train.

The mating behaviour of *E*. *heros* stinkbugs on a plant is accompanied by communication with one female (FS-1) and three different male songs (MS-1, MS-2, MRS) produced by abdomen tremulation (Fig 1). The classification of songs recorded from the plant differs from those described from mates communicating on a loudspeaker (16): MS-2 corresponds to the song determined previously as MS-3 (16); loudspeaker recorded signals classified as FS-2 and MS-2 song were not identified on a plant as special songs. Time and frequency characteristics of plant recorded songs are shown in the Table 1 and in S1 Table.

The female first song (FS-1) starts vibratory communication on the plant and triggers male responses in the calling (Fig 1a) and courtship (Fig 1b) duets. The song is characterized by pulses where regular repetition rate is modified in synchronized duets by MS-1 signals of different duration. The alternation of FS-1 with MS-2 pulse trains runs in a one-to-one manner (Fig 1b) with the rate determined by female calls. FS-1 pulse frequency increases with signal duration (Fig 1a and 1b). The male first song (MS-1) is the regular response to FS-1 in the calling phase of mating behaviour (Fig 1a). The song is composed of about seven seconds-long pulses whose duration did not significantly vary individually (n = 8) (One-way ANOVA, F = 2.055, df between groups = 7, df within groups = 151, P = 0.0521,) in contrast to their repetition time and frequency values (One-way ANOVA, F = >8.5, df = 7, P<0.0001). MS-1 pulses are characterized by frequency decrease with time along the pulse duration. The male second song (MS-2) was recorded in the courtship phase of mating behaviour, when potential mates were close to each other. The MS-2 song is composed of pulse trains emitted in the intervals between consecutive FS-1 pulses, resulting in one-to-one alternation with triggering signals (Fig 1b). The pulse train duration differed significantly between 6 tested males (One-way ANOVA, F = 21.03, df between groups = 5, df within groups = 104, P<0.0001), ranging between individual minimal and maximal means of 1455.3 ± 429.2 (N = 20) and 3181.5 ± 941.1 (N = 20) ms. The frequency characteristics of the MS-2 pulse trains differ from those of FS-1 and MS-1 pulses by their broad spectra and by lack of frequency modulation (Fig 1). Prior to copulation the female stops singing and males change the MS-2 song to a continuous sequence of pulses recorded minutes long when in copula. Significant differences between individuals were measured for MS-2 pulse time and frequency parameters (One-way ANOVA, F = >3.1, df = 7, P<0.01). The male rival song (MRS) (Fig 1c) was recorded when two males called and courted the same female. The rival duet is characterized by one-to-one alternation of MRS pulse trains without overlapping and inhibits emission of calling and courtship songs. MRS pulse trains have a complex pulse frequency modulation pattern, with periods of rapid frequency decrease separated by those with flat frequency level (Fig 1c). The frequency and time characteristics of MRS signals analyzed in 5 pairs of males differ significantly (One-way ANOVA, F = >6.6, df = 4, P<0.0001).

### Interference of overlapped signals

**Fig 2:**
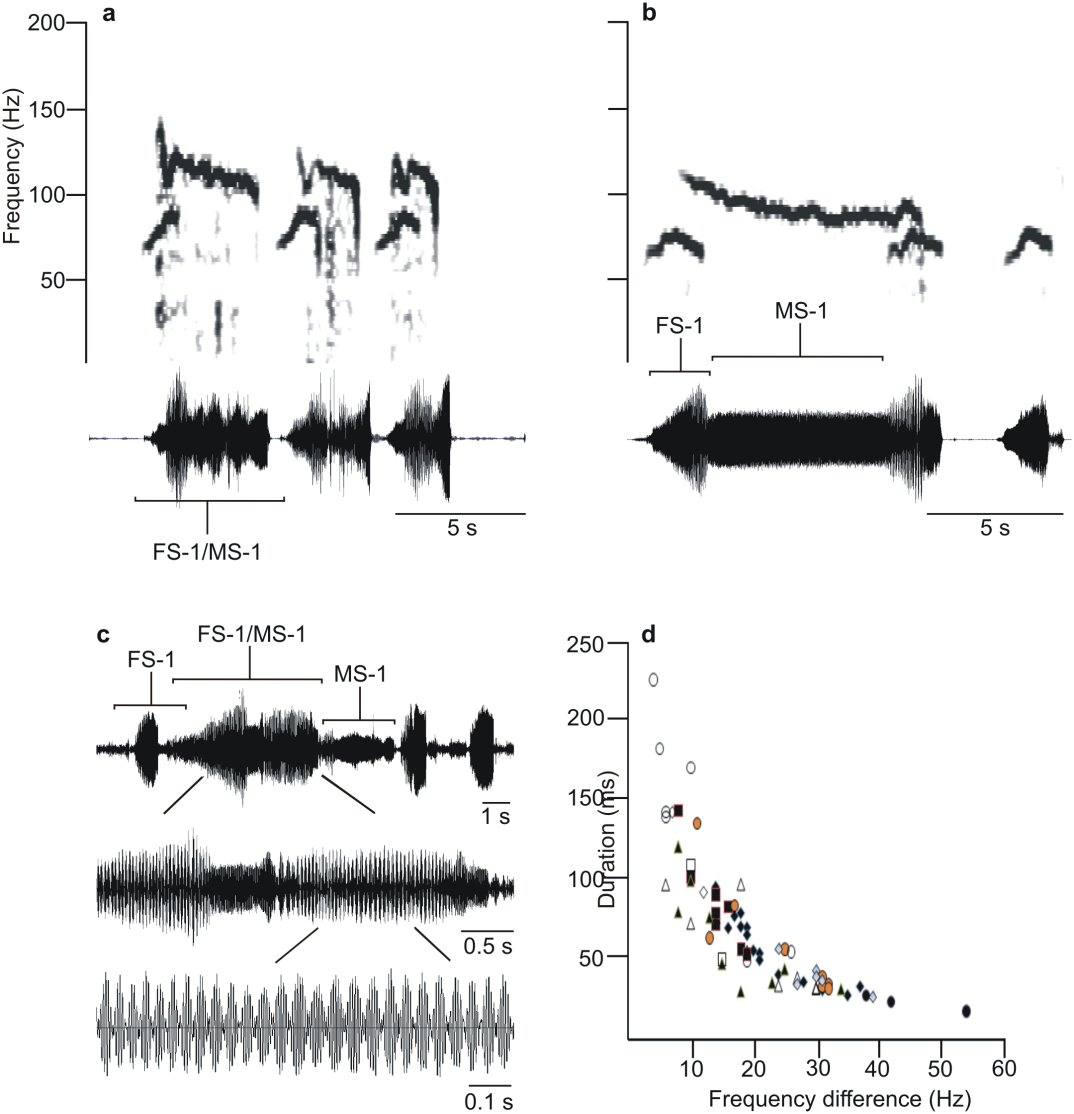
Sonagrams and oscillograms of overlapped FS-1 and MS-1 pulses of *Eushistus heros* that show a relation between interference pulse duration and frequency difference between masked signals. a, b: overlapped FS-1 and MS-1 pulses. c: overlapped FS-1 and MS-1 signals shown with different time axes. d: mean duration of pulses recorded in naturally emitted FS-1/MS-1 overlapped regions. Means were calculated for 10 different males with 2–7 MS-1 signals analyzed in each. Single mean duration values were calculated in 3 to 34 pulses within each signal. SD values were below 40% of the mean value. Regression (type power) equation: y = 814.16x^-0.911^, R^2^ = 0.8521.

Overlapping of calling signals was recorded when FS-1 pulses were longer than MS-1 response latency (Fig 2a) and/or when MS-1 pulses of longer duration than the FS-1 pulse repetition time did not inhibit the following FS-1 signal emission (Fig 2b). Overlapped FS-1/MS-1 vibrations induced interference characterized by regularly repeated fused pulses (Fig 2c) whose duration increased with decreased difference between frequencies of MS-1 and FS-1 vibrations (Fig 2d). Interference does not occur in the alternation of overlapped narrow-band frequency modulated FS-1 pulses with broad-band MS-2 pulse trains that takes place in courtship.

**Fig 3:**
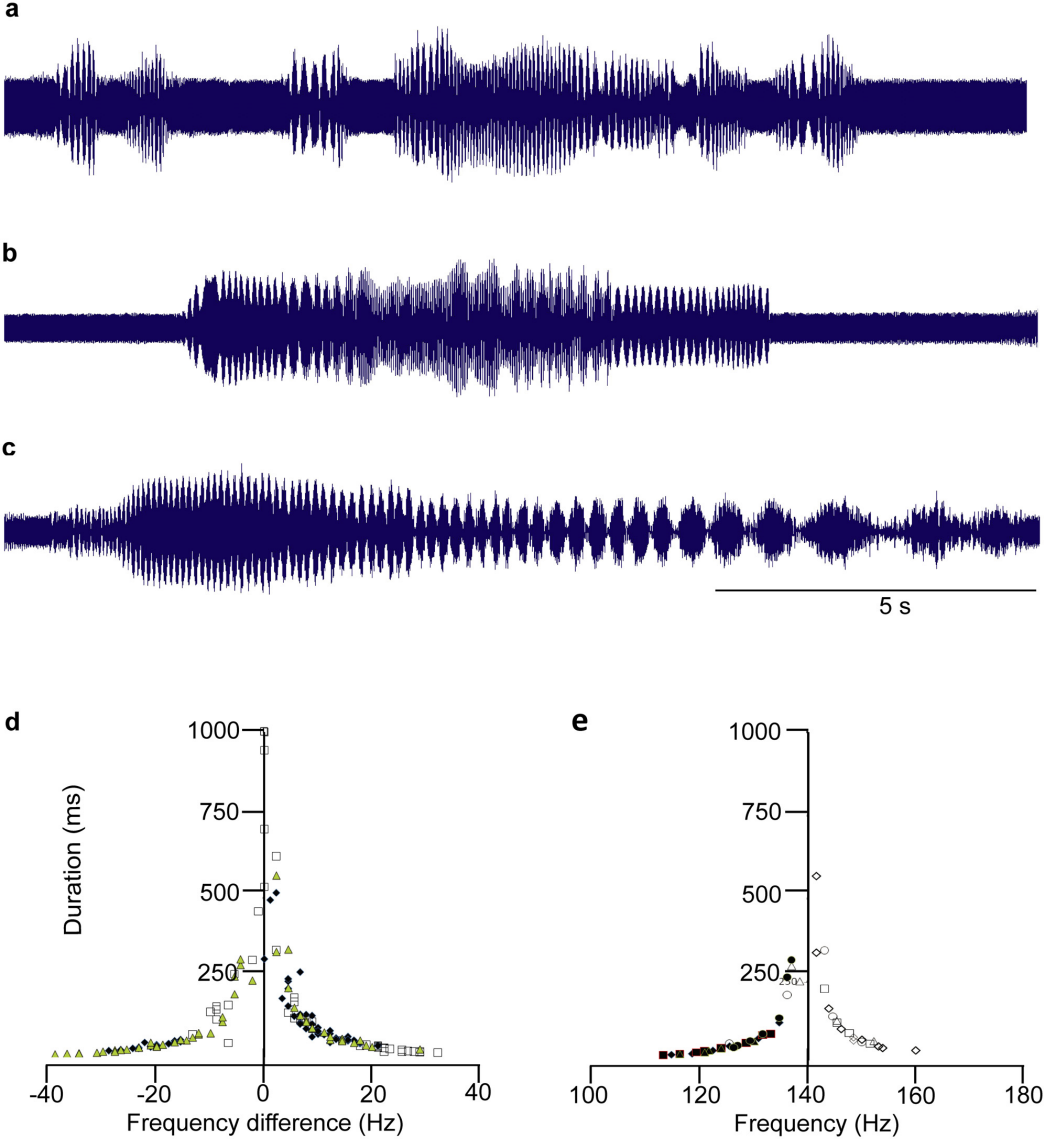
Naturally emitted FS-1 and MS-1 pulses overlapped by continuous pure tones. **The relation between interference pulse duration and the difference between MS-1 and pure tone frequencies at different frequency and velocity levels**. a,b, c: naturally emitted FS-1 and MS-1 signals overlapped by (a) 100 Hz/0.69 mm/s, (b) 125 Hz/1.13 mm/s and (c) 150 Hz/0.60 mm/s pure tones. d: mean interference pulse duration (SD<40% of the mean value, N = 3–23, n = 6) of naturally emitted MS-1 signals overlapped by 100 Hz/0.22 mm/s (black diamonds), 125 Hz/0.22 mm/s (open squares) or 150 Hz/0.22 mm/s (grey triangles) pure tone vibration. Means were determined in 1000 ms sections beginning from start to end of the MS-1 signal, e: mean pulse duration (SD<40% of the mean value, N = 3–26, n = 9) of naturally emitted MS-1 signals on soybean masked by 150 Hz/0.343–1,151 mm/s pure tone vibration. Black markers: MS-2 signals with FM decreasing from a starting dominant frequency below 150 Hz; open markers: MS-2 signals with starting frequency of FM above 150 Hz. Means were determined in 1000 ms sections beginning from the start to the end of the MS-1 signal. Time bar 5 seconds.

**Fig 4:**
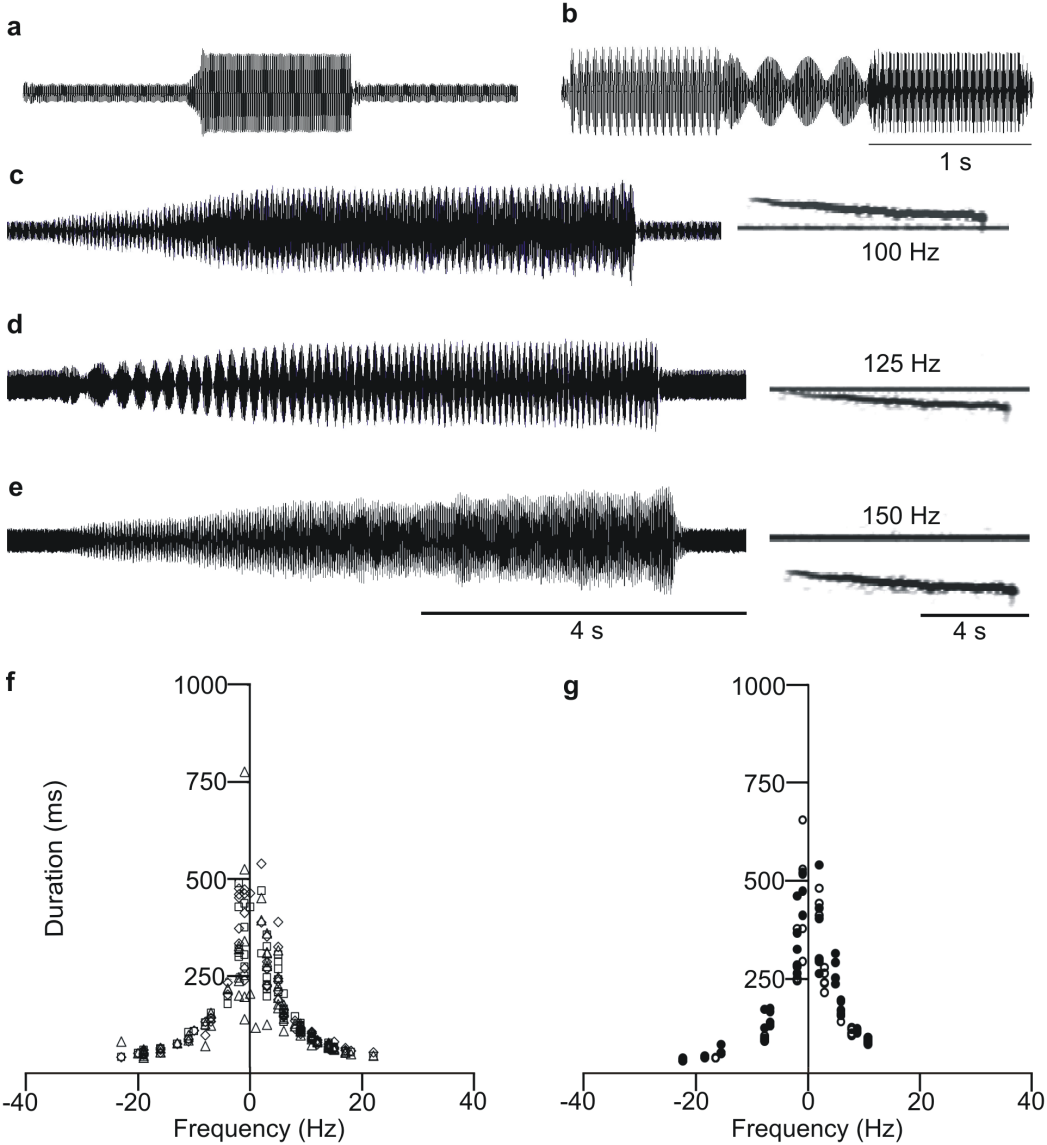
Interference induced on a soybean plant by overlapping pure tones and playback MS-1 signals. Oscillograms of a continuous 150 Hz/1.26 mm/s vibration masking (a) a 150 Hz/5.29 mm/s pulse and (b) fused 125 Hz/3.85 mm/s (left), 150 Hz/2.69 mm/s (middle) and 200 Hz/3.49 mm/s (right) pulses. Oscillograms (left) and sonograms (right) of MS-1 (dominant frequency = 111 Hz, velocity = 7.46 mm/s) playback overlapped by (c) 100 Hz/1.47 mm/s, (d) 125 Hz/1.48 mm/s and (e) 150 Hz/1.88 mm/s continuous vibration. f: mean (N = 2–24) pulse duration of MS-1 signals (n = 12) (SD<40% of the mean) masked by 125 Hz/1.16 mm/s pure tone and recorded on soybean (diamonds), *C*. *cayan* (squares) and bean (triangles); pulse duration was determined in 12 play-back signals in 1000 ms sections starting from the beginning to the end of the MS-1 signal. g: mean (N = 2–24) pulse duration of two artificially induced MS-1 signals (SD<40%) masked by 125 Hz pure tone of different velocities and determined in 1000 ms sections, starting from beginning to of the MS-1 signals recorded on soybean.

Vibration of the plant with continuous pure tones did not change the general pattern of communication, but triggered production of MS-1 and MS-2 pulses at frequencies of 100 and 125 Hz, inducing interference with overlapped naturally emitted vibratory signals (Fig 3a–3c). The inverse relation between the duration of interference pulses and the frequency difference of masked signals did not depend either on the pure tone frequency (Fig 3d) or velocity (Fig 3e). Interference was also induced by play-back vibration of the plant by two pure tones (Fig 4a and 4b) or by one pure tone and pre-recorded MS-1 signals (Fig 4c–4e). Inverse relations between interference pulse duration and the difference between frequencies of overlapped play-back vibrations were observed on different plants (Fig 4f) and at different continuous pure tone velocities (Fig 4g). The duration of pulses induced by interference ranged in masked pure tones (Fig 4a and 4b) between 202.7 ± 7.5 ms (N = 15, n = 5) at the 6 Hz difference and 19.7 ± 0.7 ms (N = 50, n = 5) at the 50 Hz difference. A pulsed amplitude modulation (AM) pattern was not induced in overlapped pure tones of the same frequency. Interference pulse duration did not depend significantly either on the tested background pure tone frequency (one way ANOVA, F = 0.2224, df between groups = 3, df within groups = 296, P = 0.8808) or on velocities ranging from 0.2 to 1.9 mm/s.

### Strategies to avoid interference

**Fig 5:**
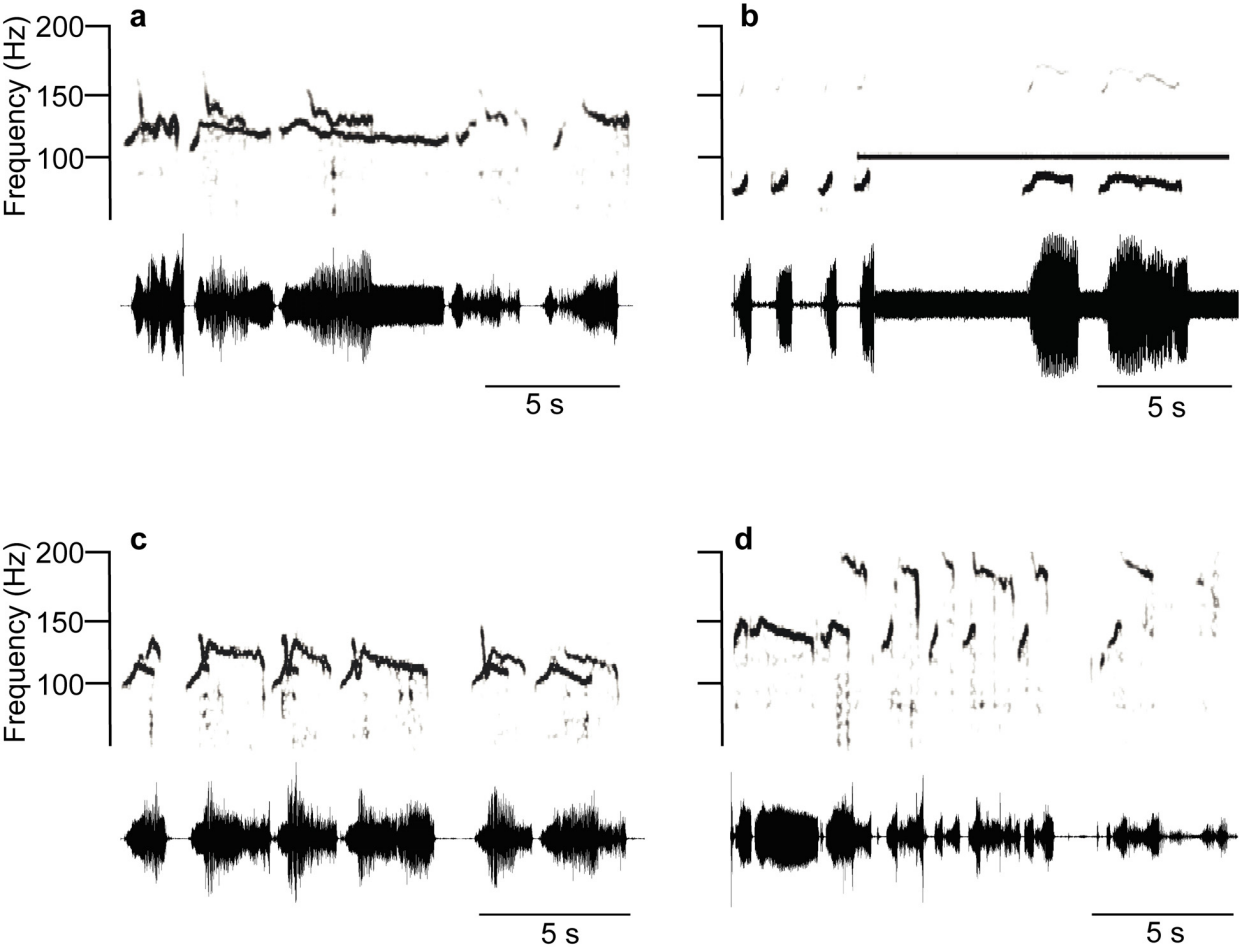
Changes of overlapped *Eushistus heros* FS-1 and MS-1 pulse time and frequency characteristics. Sonograms (above) and oscillograms (below) of (a) increased FS-1 pulse duration when overlapped by MS-1 signals or (b) by 100 Hz/0.69 mm/s pure tone. c: the U-shaped FM sweep of MS-1 pulses masked by FS-1 signals. d: the increased frequency and decreased duration of MS-1 pulses in a duet with FS-1 signals.

The high variability of the FS-1 pulse repetition time and of the MS-1 pulse duration (Table 1) gives *E*. *heros* females and males the possibility of avoiding mutual overlapping by synchronization of time parameters in calling duets (Fig 1a). Outside the limited range of time parameter adaptation, signals overlap each other and induce interference, which significantly changes their amplitude modulation pattern (Fig 2). Females and males react differently to minimize the consequences of signal overlapping. Females increase pulse duration and modify the frequency modulation (FM) pattern of the prolonged signal to run in parallel with that of overlapping MS-1 pulse (Fig 5a). The same reaction was achieved by vibrating the plant with a continuous pure tone (Fig 5b): FS-1 pulse duration was increased significantly at vibration with 100, 125 and 150 Hz pure tones (Table 2). Males respond to signal overlapping by changing their FM pattern at the start of the MS-1 signal (Fig 5c) and by increasing the frequency difference between MS-1 and FS-1 pulses (Fig 5d). When FS-1 and MS-1 are overlapped by each other, males produce a characteristic U-shaped sweep of frequency decrease and increase per time (Fig 5c). The U-shaped MS-1 FM pattern was analysed in 8 different pairs and the tested parameters (Table 3) varied significantly between them (One-way ANOVA, F>3.4, df = 7, P<0.001).

**Table 2:** *Euschistus heros* FS-1 pulse duration in control and overlapped conditions.

Pure tone		FS-1 duration (ms)	
frequency (Hz)	velocity (mm/s)	Control N/n	Overlapped N/n
75	0.28–1.31	1118.1±253.8 (60/2)	1083.1±258.0 (60/2)
100	0.21–2.01	1281.7±308.3 (60/2)	2745.5±1326.0 (60/2)***
125	0.18–1.1	1071.8±184.7 (40/2)	1565.3±300.9 (40/2)***
150	0.86–2.57	1221.5±227.2 (60/2)	1388.3±236.3 (60/2)***
200	0.07–0.55	1183.0±197.6 (50/2)	1169.0±173.0 (50/2)

Means and standard deviations are shown with number of signals (N) and number of animals (n).

*** Significant difference (p<0.0001) between means in control and overlapped conditions (two-tailed *t*-test for unpaired data).

**Table 3:** Frequency characteristics of *E*. *heros* overlapped FS-1 and MS-1 pulses measured in 8 different couples.

Parameter	Frequency (Hz)	Frequency (Hz)
FS-1 start frequency	94.6 ± 7.1 (N = 10)	121.4 ± 4.5 (N = 17)
MS-1 start frequency	130.3 ± 4.6 (N = 12)	170.9 ± 6.1 (N = 17)
FS-1/MS-1 start frequency difference	21.8 ± 6.2 (N = 12)	48.5 ± 7.7 (N = 40)
FS-1 upward frequency sweep	4.3 ± 6.5 (N = 20)	11.4 ± 6.4 (N = 20)
MS-1 downward frequency sweep	6.0 ± 5.8 (N = 12)	29.2 ± 15.1 (N = 17)
FS-1/MS-1 lowest frequency difference	-7.6 ± 9.1 (N = 20)	18.2 ± 7.8 (N = 20)
MS-1 upward oriented sweep	12.3 ± 5.7 (N = 12)	28.3 ± 10.5 (N = 17)

Minimal and maximal means with standard deviations are shown when differences among individuals were significant: One-way ANOVA, F>5.699, df = 7, P>0.0001) N, number of signals analysed for each individual.

## Discussion

Recording the vibratory communication signals of *E*. *heros* stinkbugs during mating on a plant revealed characteristic calling, courtship and rivalry duets constituted by one female (FS-1) and three different male songs (MS-1, MS-2, MRS). Irregularly repeated several second-long pulses with a complex amplitude modulation pattern [16] were often recorded on the plant as overlapped female and male calling signals. The role of the high amplitude vibrations produced by tremulation of the whole body or by wings “buzzing” is not clear although, these signals could enable interplant communication by their airborne component [21] as was shown in leafhoppers by Eriksson and co-workers [22]. *E*. *heros* females and males produce, by tremulation of the abdomen, signals with species and gender specific times and extensive frequency modulation parameters. Although several other pentatomine species, like *E*. *conspersus* (Uhler) [23], *Piezodorus lituratus* [24], *Chlorochroa uhleri*, *C*. *ligata* and *C*. *sayi* [25], emit frequency modulated signals, the dispersive nature of the corrected bending waves [4, 5] does not favour their use in long range communication through stems and other rod-like plant structures. Resonance [8] causes frequency dependent, cyclic variation of signal intensity with distance [26], which changes the amplitude pattern of frequency modulated vibration, as shown for artificially induced synthesized 40–160 Hz FM [2] and for naturally emitted *N*. *viridula* signals [26]. *E*. *heros* males and females change both the frequency level and the frequency modulation pattern in response to the communication signals of the duetting partner. This leads to the conclusions that frequency is important in determining the characteristics and efficiency of vibratory signal transmission through plants [5, 9] and is also involved indirectly in mate recognition and location processes. The level of signal species and gender specificity that is mediated mainly by relatively stable time characteristics [15, 27, 28, 29, 30] is thus enlarged. This hypothesis is supported by an earlier study in male *P*. *lituratus* that responds with different songs to play-back of conspecific male rival song signals with changed FM characteristics [18]. The significant influence of the sound and vibratory environment on the efficiency of mate location and recognition is demonstrated in the recent review on vibrational communication networks by Virant-Doberlet and co-workers [14]. Low frequency vibrations produced by wind and/or water drops have only a minor effect on the signal-to-noise ratio of stinkbugs that communicate through a narrow frequency window. On the other hand masking signals with characteristic narrow band, low frequency spectral properties constitutes an obstacle to extracting relevant information on the emitter’s location and identity. Male *N*. *viridula*, for example, discriminate between conspecific female calling song signals and those of the alien stinkbug species *Acrosternum hilare* (Say) in a sequence of non-overlapped pulses, but make significant errors of orientation and species recognition when hetero- and conspecific signals overlap each other [30]. Overlapping reduces the directional information in a treehopper, *Tylopelta gibbera* (Fowler) (Hemiptera: Membracidae) [31] and disrupts reproductive behaviour in *Scaphoideus titanus* [22, 32]. Rodriguez and Barbosa [33], in a review on *Euchenopa* treehopper vibrational duetting, showed the potential influence of signals of one gender on signalling behaviour of the other. The *Euchenopa* treehopper female, for example, changes the time characteristics of their duetting signals according to the level of attractiveness of male emissions [34, 35]. Males respond better to female duetting signals recorded as a response to stimuli they find attractive. Species identity cues are less important in the latter case. To our knowledge, similar studies have not been conducted in Pentatomidae. Mutual overlapping of stinkbug duetting mating signals occurs when the adaptation range of their time parameters does not allow emission in silent periods between consecutive calls. Calling signals of *E*. *heros* overlap and fuse into a complex combined vibration with frequency dependent pulsed amplitude pattern induced by interference. The main strategy of brown stink bug males and females is to increase the difference between the frequencies of overlapped vibrations, and thus to decrease the duration and increase the repetition rate of pulses produced by interference. Females modify the FM pattern of prolonged calling pulses to run in parallel with those of overlapped MS-1 pulses and males change the frequency level of their responses below or above the value of the overlapping signal. The hypothesis that decreasing pulse duration by frequency modification of male and female overlapped signals minimizes the effect of changed amplitude pattern is supported by the study of Miklas and co-workers [36] on male *N*. *viridula* responsiveness to two types of conspecific female calling song signals. Pulse trains of both types have similar duration and repetition rates but differ in the number of pulses per train; first type pulse trains (non-pulsed type) contain a short pre-pulse followed by a long one and those of the second, pulsed type, are composed of rapidly repeated short pulses [37]. On a non-resonant loudspeaker membrane significantly more responses were recorded from male stimulated with the non-pulsed than with the pulsed type of female calling song. On the plant, male responses to the two types of female calling song did not differ significantly. Rapidly repeated pulses within the pulse train of the second type were shown to fuse during transmission through a plant, to the extent that single pulses are not recognized as units and the pulse train is not discriminated from long signals of the first type. Recording of vibratory emissions on a non-resonant substrate enables species and gender specific time and frequency characteristics of signals to be compared under the same conditions, but limits behaviour to artificial and restricted space. Plants with different mechanical properties change transmitted vibratory signal characteristics differently and, consequently, demand tuning of mate reactions to preserve the information. The work of Miklas and co-workers [36] and our present study show that investigations on non-resonant and resonant substrates are needed to obtain a clearer picture of processes of communication with vibratory signals. The repetition rate of interference induced pulses ranges between 5 Hz at low and 100 Hz at high frequency difference. The system of low frequency vibratory receptor neurons in the related pentatomine species *N*. *viridula* [11] responds in a phase coupled manner below 100 Hz with a sensitivity that decreases with increasing frequency. The increasing repetition rate of interference pulses decreases the sensory input from the low frequency receptors and consequently decreases the impact on the input from the middle frequency receptor neurons of best sensitivity above 100 Hz. This interplay of low and middle frequency sensory systems needs to be experimentally confirmed by neurophysiological studies at the receptor and higher order neuronal levels. We can conclude that overlapping of duetting signals of similar frequency characteristics induces interference characterized by a frequency dependent, pulsed amplitude modulation pattern. *E*. *heros* males and females react by changing the time and frequency characteristics of their calling song signals to avoid, or minimize, the time and amplitude pattern changes caused by interference and to favour the intra-specific communication. Frequency level and FM pattern changes confirm the hypothesis that frequency is important, not only in insect-plant interaction during transmission of vibratory signals, but also enlarges the range of signal adaptations to environmental noise of biotic or abiotic origin within or outside the communication frequency range. Detailed studies are needed to obtain answers to questions concerning behavioural and vibratory responses to interference and to changed parameters of overlapped signals. Reinvestigation of previously recorded and analyzed vibratory communication signals in Pentatomidae is needed to confirm or reject the hypothesis that duetting with narrowband, low frequency signals at similar frequency levels demands precise timing to avoid overlapping. Furthermore, the responses of receptor and higher order vibratory neurons to overlapped signals and to frequency modulation in general have yet to be been investigated in Pentatomidae.

## Supporting Information

S1 Table Temporal and frequency properties of *Eushistus heros* substrate-borne FS-1, MS-1, MS-2 and MRS signals. (DOC)
