# Supplementary material for: Interference of Overlapping Insect Vibratory Communication Signals: An Eushistus heros Model
Source: PLoS One. 2015 Jun 22;10(6):e0130775. doi: 10.1371/journal.pone.0130775 (PMC4476573; doi:10.1371/journal.pone.0130775)
Supplement: S1 Table — (DOC) [file pone.0130775.s001.doc]

**Table 1 (extended).** Temporal and frequency properties of *Eushistus heros* substrate-borne FS-1, MS-1, MS-2 and MRS signals.

|  |  |  |  |  |  |
| --- | --- | --- | --- | --- | --- |
|  |  |  |  |  |  |
| **Song type** | **FS-1a (N=20)** | **FS-1b (N=20)** | **MS-1 (N=13^x^,20)** | **MS-2 (N=25,50)** | **MRS (N=20)** |
| **Duration (ms)** | 817.4±67.8 1164.5±178.9  1170.8±170.4  1206.6±204.0  1381.5±150.3 | 924.5±186.0  1073.4±106.3  1347.3±199.3  1520.8±225.1  1605.0±295.0 | 6022.3±1064.7**^x^**  6148.0±1127.2  6462.6±855.6  6570.4±1978.7  6770.2±1440.9  7359.7±2513.4  7521.9±2047.4  7572.9±2510.4 | 57.9±9.1*  60.9±9.6*  79.3±15.6*  81.8±15.9*  71.3±16.2**  78.6±11.6**  87.3±27.3**  87.8±13.0**  92.8±13.4**  94.4±23.2**  115.3±8.7***  116.6±13.4***  118.8±22.3***  130.5±19.3*** | 735.2±225.1  993.9±123.0  1243.5±108.2  1391.6±1914.6  1403.8±382.4 |
| **Repetition time**  **(ms)** | 2748.0±718.4  2915.3±702.3  2939.7±624.8  2946.8±677.7  5014.0±1607.3 | 2475.4±328.7  2658.9±420.9  2743.1±424.2  3076.5±895.1  3097.6±534.5 | 16104.0±9103.0  16786.2±3545.9  18723.8±7968.8  19485.5±5129.4  20641.1±11804.7  24602.0±10240.0  25969.5±9297.7 **^x^**  34069.0±8859.0 | 140.7±39.5**  140.8±38.2**  145.2±31.8**  149.9±54.3**  153.8±32.7**  175.6±77.5**  161.8±17.9***  166.9±20.4***  225.6±35.3***  288.8±50.7*** | 998.0±185.0  1273.1±93.2  1419.4±82.2  1609.3±178.4  1853.1±492.8 |
| **Dominant frequency**  **(Hz)** | 111.7±1.7  112.1±1.6  123.5±3.6  124.7±4.9  129.7±3.9 | 107.4±4.1  112.3±22.9  117.9±1.7  123.5±3.4  134.4±1.7 | 106.9±3.6  124.8±8.0  129.5±5.5  133.3±5.1  143.1±10.7  145.4±9.8  147.0±9.8  158.7±3.7 **^x^** | 109.8±2.1*  124.4±16.5*  136.0±5.6*  147.1±4.9*  110.9±3.5**  126.0±16.3**  136.6±4.4**  141.5±3.0**  150.9±8.4**  152.3±1.3**  105.0±3.3***  105.3±1.7***  119.4±21.0***  153.3±10.2*** | 108.3±3.0  108.3±1.6  108.9±2.3  123.5±4.5  136.4±7.1 |
| **Frequency FM-start (Hz)** | 102.3±3.3  108.2±7.7  111.9±5.2  116.6±3.3  119.7±8.7 | 105.4±2.3  111.9±4.1 113.1±2.5  119.0±4.3  133.4±3.2 | 118.8±3.6  140.3±2.7  140.5±3.5  164.1±5.7  164.7±3.3  174.3±4.1  176.8±4.3  190.5±4.9 **^x^** | - | 143.7±7.2  156.2±9.7  158.5±5.0  159.0±9.6  167.2±8.7 |
| **Frequency FM-middle**  **(Hz)** | - | - | - | - | 107.8±2.4  108.2±4.9  109.8±3.0  123.9±4.7  126.6±4.0 |
| **Frequency FM-end (Hz)** | 127.1±2.5  130.5±7.3  139.4±5.6  139.5±2.4  152.0±5.1 | 119.2±6.0  125.9±3.5  126.3±6.2  127.7±3.8  144.7±4.5 | 101.5±2.9  110.8±5.7  122.8±5.7  128.6±4.5  136.5±7.2  137.1±5.9  143.3±3.0  153.5±4.8 **^x^** | - | 86.1±4.3  87.0±4.1  88.7±6.4  99.5±5.5  101.0±6.1 |
| **FM start-end (Hz)** | 19.8±8.0  22.4±13.8  24.9±3.4  27.7±5.5  35.55.3 | 8.8±5.5  11.4±4.5  13.2±5.5  13.8±5.6  14.0±5.1 | 15.4±5.5  16.7±4.9  18.8±5.2  20.8±8.0  29.8±5.9  36.7±5.9  39.1±6.1  44.9±6.6 **^x^** | - | 58.0±8.8  59.1±8.8  66.0±10.5  67.6±11.8  71.7±9.6 |
| **FM/1 s**  **(Hz/1s)** | 8.6±5.8  17.0±8.0  18.1±2.8  24.4±7.6  31.6±9.2 | 6.1±4.4  8.5±4.0  10.6±4.3  10.9±5.4  15.5±6.6 | 2.4±0.8  2.7±0.8  3.3±0.7  4.1±1.7  4.8±1.3  6.4±1.6 **^x^**  6.7±2.6  6.7±2.0 | - | 47.1±9.0  48.9±7.3  54.9±17.1  67.2±12.9  87.7±28.7 |

Individual mean (±SD) values are shown for FS-1a (female first song in a duet with MS-1), FS-1b (female first song in a duet with MS-2), MS-1 (first male song), MS-2 (second male song) and MRS (male rival song). N, number of signals analyzed for each individual, FM = frequency modulation. *MS-2 pulse train fused pulses (N=50), **MS-2 pulse train non-fused pulses (N=25), *** MS-2 pulses not grouped into pulse trains (N=50). **^x^** N=13.
